# Control over molecular motion using the *cis*–*trans* photoisomerization of the azo group

**DOI:** 10.3762/bjoc.8.119

**Published:** 2012-07-12

**Authors:** Estíbaliz Merino, María Ribagorda

**Affiliations:** 1 Instituto de Química Orgánica General, Centro Superior de Investigaciones Científicas (CSIC), C/ Juan de la Cierva, 3, 28006, Madrid, Spain; 2 Departmento de Química Orgánica, Facultad de Ciencias, Universidad Autónoma de Madrid, 28049, Madrid, Spain

**Keywords:** azobenzenes, molecular switches, nanomachines, photoisomerization

## Abstract

Control over molecular motion represents an important objective in modern chemistry. Aromatic azobenzenes are excellent candidates as molecular switches since they can exist in two forms, namely the *cis* (*Z*) and *trans* (*E*) isomers, which can interconvert both photochemically and thermally. This transformation induces a molecular movement and a significant geometric change, therefore the azobenzene unit is an excellent candidate to build dynamic molecular devices. We describe selected examples of systems containing an azobenzene moiety and their motions and geometrical changes caused by external stimuli.

## Review

This review is based on an article published in 2009 in *Anales de Química* (Real Sociedad Española de Química) [1]. Azobenzene was described for the first time in 1834 [2] and one century later, in 1937, G. S. Hartley published a study of the influence of light on the configuration of N=N double bonds [3]. The exposure of a solution of azobenzene in acetone to light allowed the discovery of the *cis* isomer. This finding was the starting point of the development of one of the best organic molecular switches described so far. The azobenzenes are organic molecules that present two aromatic rings linked by an azo group (N=N). They have properties that have led to some applications of great importance, mainly for the chemical industry. The azobenzenes are highly coloured compounds and belong to the group of so-called dyes FD&C (food, drug and cosmetics). Nowadays, azobenzene dyes represent approximately 60% of the world production of industrial dyes [4–6].

In recent years, the photochromic properties of azobenzenes have attracted great interest due to the isomerization of the N=N double bonds that occurs readily in the presence of a light source [7–9].

Like a C=C double bond, the azobenzenes have two geometric isomers (*Z*/*E*) around the N=N double bond, the *trans* isomer (*E*) is ~12 kcal∙mol^−1^ more stable than the *cis* isomer (*Z*) [10]. The energy barrier of the photoexcited state is ~23 kcal∙mol^−1^, such that the *trans* isomer is predominant in the dark at room temperature [11].

The *trans*-azobenzene easily isomerizes to the *cis* isomer by irradiation of the *trans* isomer with a wavelength between 320–350 nm. The reaction is reversible and the *trans* isomer is recovered when the *cis* isomer is irradiated with light of 400–450 nm, or heated. For many azobenzenes, the two photochemical conversions occur on the scale of picoseconds, while the thermal relaxation of the *cis* isomer to the *trans* isomer is much slower (milliseconds to days). The photoinduced isomerization of the azobenzenes leads to a remarkable change in their physical properties, such as molecular geometry, dipole moment or absorption spectrum [12–16].

**Figure 1:**
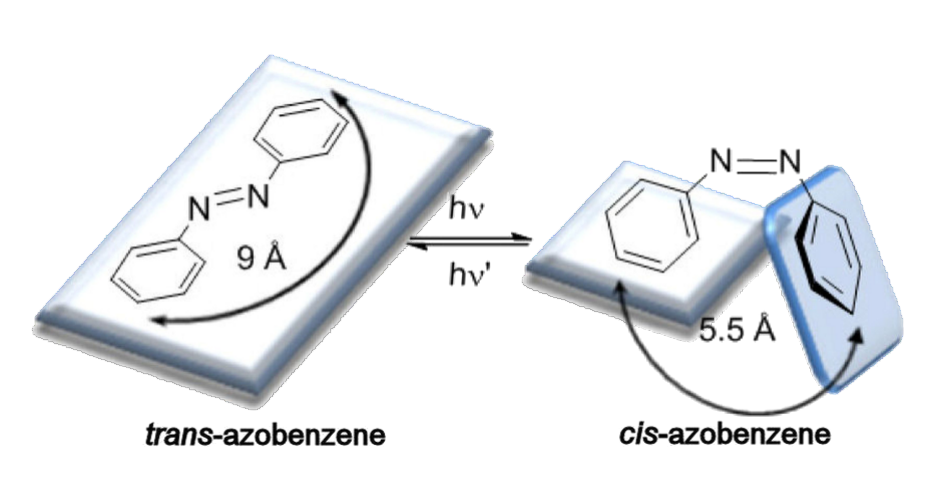
Photoisomerization process of azobenzene.

The isomerization process involves a decrease in the distance between the two carbon atoms in position 4 of the aromatic rings of azobenzene, from 9.0 Å in the *trans* form to 5.5 Å in the *cis* form (Figure 1) [17]. The *trans*-azobenzene is almost flat and has no dipole moment, whereas the *cis* isomer presents an angular geometry and a dipole moment of 3.0 D. One of the rings rotates to avoid steric repulsions due to facing of one of the π clouds of one aromatic ring to the other [9]. The arrangement of the aromatic rings is also reflected in the proton nuclear magnetic resonance spectrum (^1^H NMR). The signals of the *cis* isomer appear at higher field than the signals corresponding to the *trans* isomer, due to the anisotropic effect of the π cloud of the aromatic ring.

The UV–vis absorption spectrum of azobenzene presents two characteristic absorption bands corresponding to π→π* and n→π* electronic transitions. The transition π→π* is usually in the near UV region and is common to carbonate systems, such as stilbene [18]. The electronic transition n→π* is usually located in the visible region and is due to the presence of unshared electron pairs of nitrogen atoms [19]. Due to this second electronic transition, the dynamic photoisomerization process of azobenzenes is different to the carbonate compounds [20]. Azobenzene undergoes *trans*–*cis* isomerization by S_1_←S_0_ and S_2_←S_0_ excitations and *cis*–*trans* isomerization by exciting into the S_1_ or S_2_ state [21]. The sum of the quantum yields is different to unity, which indicates multiple pathways for isomerization. In stilbene, the isomerization occurs exclusively by rotation and the quantum yield equals unity [22].

The aromatic azocompounds are classified in three types based on the order of their energetic electronic states π→π* and n→π* [11]. This order depends on the electronic nature of the aromatic rings of azobenzene. Each type of azobenzene also has a predominant colour defined by the wavelength of the maximum absorption band (λ_max_) (indicated in brackets in each case):

1. Azobenzene type: The π→π* band is very intense in the UV region and there is one n→π* weaker in the visible (yellow colour). The electronic nature of the aromatic rings is very similar to simple azobenzene (Ph–N=N–Ph).
2. Aminoazobenzene type (*o-* or *p*-(X)–C_6_H_4_–N=N–Ar): The π→π* and n→π* bands are very close or collapsing in the UV–vis region. In this case, the azocompounds have electron-donor substituents (X) in the *ortho* or *para* positions (orange colour).
3. Pseudo-stilbene type [(X)–C_6_H_4_–N=N–C_6_H_4_–(Y)]: The absorption band corresponding with π→π* transition is shifted to red, changing the appearance order with respect to the band n→π*. The azocompounds of this type present donor substituents (X) and electron acceptors (Y) at the 4 and 4' positions, respectively (push/pull system) (red colour).

**Figure 2:**
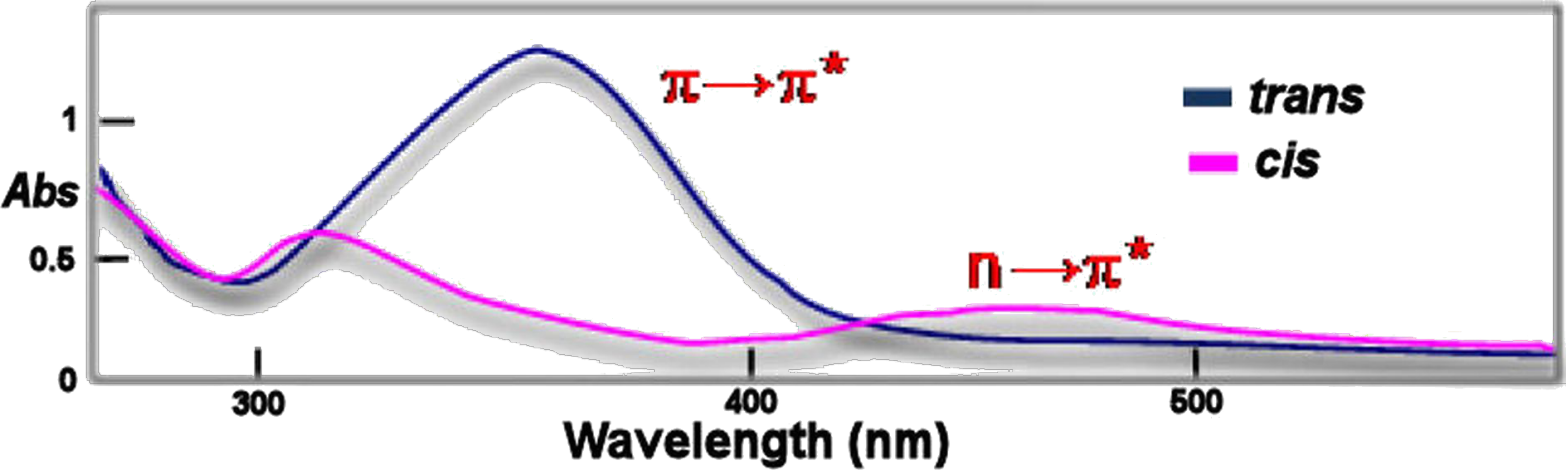
Representative example of an UV spectrum of an azocompound of the azobenzene type (blue line: *trans* isomer; magenta line: *cis* isomer).

The isomerization process normally involves a colour change to more intense colours. The absorption spectra of both isomers differ mainly in the following aspects (Figure 2) [23]:

*Trans* isomer: The absorption band π→π* is very intense, with a molar extinction coefficient (ε) ~ 2–3 × 10^4^ M^−1^∙cm^−1^. The second band (n→π*) is much weaker (ε ~ 400 M^−1^∙cm^−1^) as this transition is not allowed in the *trans* isomer by the symmetry rules.

*Cis* isomer: The absorption band π→π* is shifted to shorter wavelengths (hypsochromic effect) decreasing significantly in intensity (ε ~ 7–10 × 10^3^ M^−1^∙cm^−1^). The electronic transition n→π* (380–520 nm) is allowed in the *cis* isomer, resulting in an increase in the intensity (ε ~ 1500 M^−1^∙cm^−1^) with respect to the *trans* isomer.

**Figure 3:**
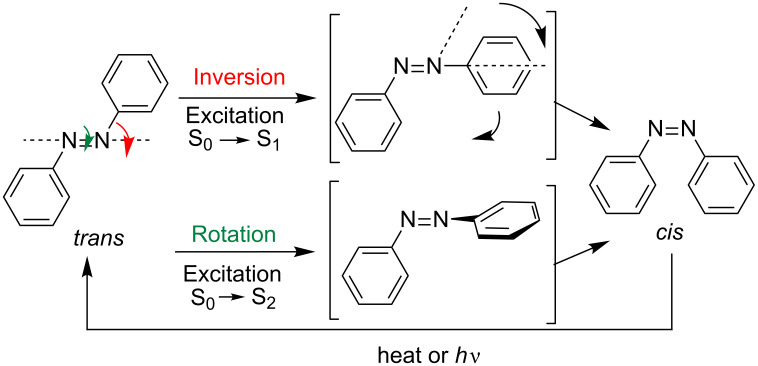
Mechanistic proposals for the isomerization of azobenzenes.

These differences allow carrying out a photochemical interconversion by irradiation with light of a certain wavelength, obtaining different proportions of the *cis* and *trans* photostationary states. The excitation caused by the wavelength is dependent on the nature of the substituents of the aryl groups. In most cases, *trans*→*cis* isomerization is promoted by irradiation with wavelengths between 320–380 nm, while exposures to λ ~ 400–450 nm favor the *cis*→*trans* photoreversion. The mechanism is not well established. Several mechanistic studies have been performed on the isomerization reversal route *cis*→*trans* of azobenzene to investigate the effect of the substituents on the benzene rings as well as the influence of several parameters [24–27]. The available data suggest that the isomerization of azocompounds can proceed through the reversal of one of the N–C bonds or by the rotation of the N=N double bond. The nonbonding electron pair of each nitrogen atom may lead to one n→π* electronic transition (S_0_→S_1_) with inversion at the nitrogen atom (inversion mechanism) [28–29]. On the other hand, the isomerization can also occur through a rotation mechanism [11,30], which involves a π→π* transition (S_0_→S_2_) (Figure 3). This mechanism is similar to that produced in the isomerization of stilbene [23].

### Azobenzenes as molecular switches

A molecular switch is a molecular system that allows mechanical movements to be carried out when the system is subjected to an external stimulus, such as light, resulting in conformational and environmental changes of the switch.

The basis of a molecular switch is the reversible transformation of chemical species caused by light between two states of a molecule with different absorption spectra.

These photoisomerization processes modify the absorption spectra and can produce variations in different physicochemical properties of molecules, such as ion complexation, refractive index, electrochemical behaviour, and very significant conformational changes in polymers. There may also be variations in the organization of large assemblies of molecules in gels or liquid crystals. When polarized light is used, the photoisomerization often induces a reorganization of chromophores that can be reflected in the circular dichroism spectra.

The basic condition for a molecule to behave as a switch is the existence of two different and stable isomeric forms that interconvert when an external stimulus is applied to it.

The most important requirements for a molecule to behave as a molecular switch are the following [31–35]:

1. The transformation between the two interconvertible structures that comprise the molecular switch must be produced easily and selectively by irradiation with light of a certain wavelength.
2. The thermal interconversion between two isomers should not occur in a wide temperature range, thus allowing the storage of information for an almost infinite length of time.
3. The isomers should have an appreciable resistance to fatigue (number of cycles without decomposition), with the possibility to carry out the cycle of write/erase several times, and should not cause thermal degradation or photochemical side-products.
4. The two structural forms should be easily detectable.
5. An efficient interrupt process. High quantum yields have to be achieved by using short irradiation periods.
6. The response times have to be quick to be also fast interrupting cycles.
7. It is necessary that all properties remain unchanged when the compound used as the photoswitch is a part of a macromolecular structure.

A variety of photosensitive devices, such as smart polymers [36–37], liquid crystals [38–39], intelligent enzymes [40], and various switches and molecular machines [41–48], have been developed by using the photochromic properties of azobenzene and benefiting from its easy synthesis [4,6,49–54]. The molecular motion that occurs in the photoisomerization has allowed the development of azo-structures that have grown in complexity, originality and usefulness [55–58]. This review includes some of the most outstanding examples.

### Photoisomerization of azobenzenes: A simple molecular motion

The introduction of an azobenzene fragment in a molecule with biological activity [59–63], such as a protein, can allow the spatial and temporal control of a variety of biological processes through illumination, by means of the direct regulation of enzymatic activity [64–67], peptides, proteins, nucleic acids [68–76], receptors [77–82], or ion channels [83–85], or by modulation of the concentration of several labelled molecules. This strategy is very attractive because it allows control over the conformation and consequently the activity of biomolecules in a reversible way without the addition of any reagent. Structural effects caused by the isomerization can be amplified in the host or initiate a cascade of photophysical and photochemical secondary responses. The first application of an azobenzene in biology was published in the late 1960s and was used to photoregulate the activity of chymotrypsin, a digestive enzyme [86]. Later, a similar strategy was applied in functional and structural studies of the acetylcholine receptor of nicotinic type [87]. The *trans*→*cis* photoisomerization of 4,4'-trimethylammonium methyl substituted azobenzene produced an increase in the concentration of acetylcholine agonists as a result of the specific interaction of both isomers with the acetylcholine receptor that is present in excitable membranes. In this way, it is possible to control the permeability changes, allowing the ion motion during the generation of the bioelectric impulse.

**Figure 4:**
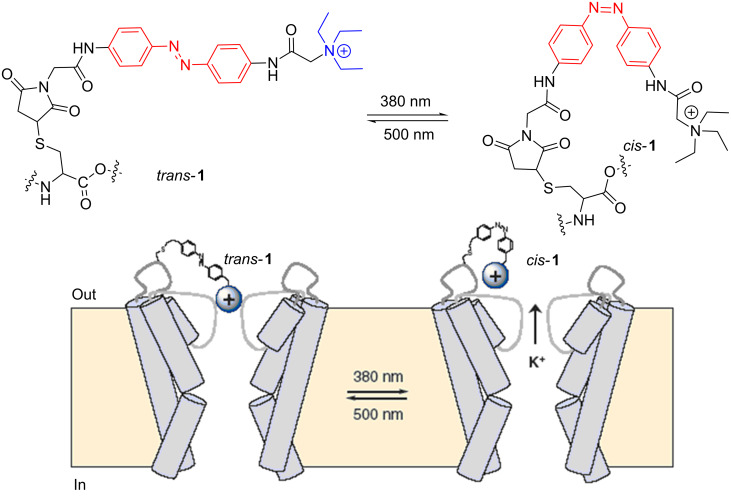
Representation of the photocontrol of a K^+^ channel in the cellular membrane based on the isomerization of azocompound **1**. Reprinted (adapted) with permission from Macmillan Publishers Ltd: *Nat. Neurosci.* **2004**, *7*, 1381–1386, copyright (2004).

The isomerization of azocompounds has been used as a synthetic tool to control the opening and closing of pores in cellular membranes, essential for the transport of ions. An illustrative example is described by Trauner, Kramer and co-workers to control the K^+^ channels in neuronal cells (Figure 4) [88].

**Figure 5:**
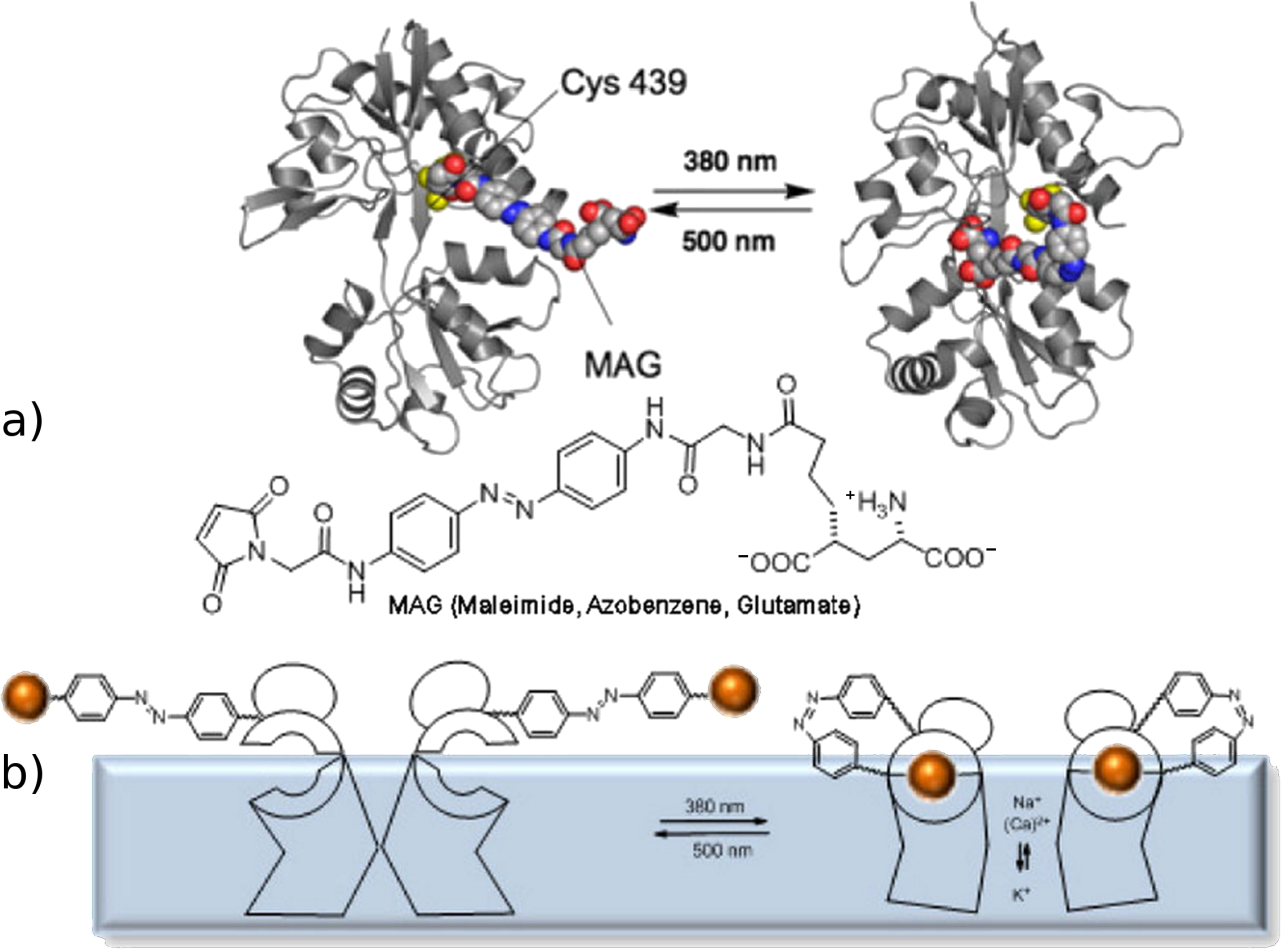
(a) MAG interaction with iGluR; (b) photocontrol of the opening of the ion channel by *trans*–*cis* isomerization of azobenzene. Reprinted with permission from Macmillan Publishers Ltd: *Nat. Chem. Biol.* **2006**, *2*, 47–52, copyright (2006).

The azobenzene **1** is a terminal quaternary ammonium salt, thus when **1** adopts the *trans* configuration, the flow of K^+^ ions is blocked. After irradiation with λ = 380 nm, the *cis* isomer brings the aromatic rings closer, shortening its length, and hence the channel blocking is inefficient, allowing the passage of ions. The development of systems capable of photoregulating the activity of ion channels is extremely important in neurobiology. Recently, a maleimide, azobenzene and glutamate derivative (MAG) was used as a photochromic agonist of an ionotropic glutamate receptor (iGluR) (Figure 5a) [89–91]. The chromophore consists of a terminal maleimide unit, which is associated covalently to the protein via a cysteine residue, a central azobenzene unit and a glutamate head. Only the *cis* form of the azobenzene allows the approach of the fragment and the interaction of glutamate with the active site of the protein. When this interaction occurs, the protein folds as a clamshell, triggering the opening of the ion channel (Figure 5b).

**Figure 6:**
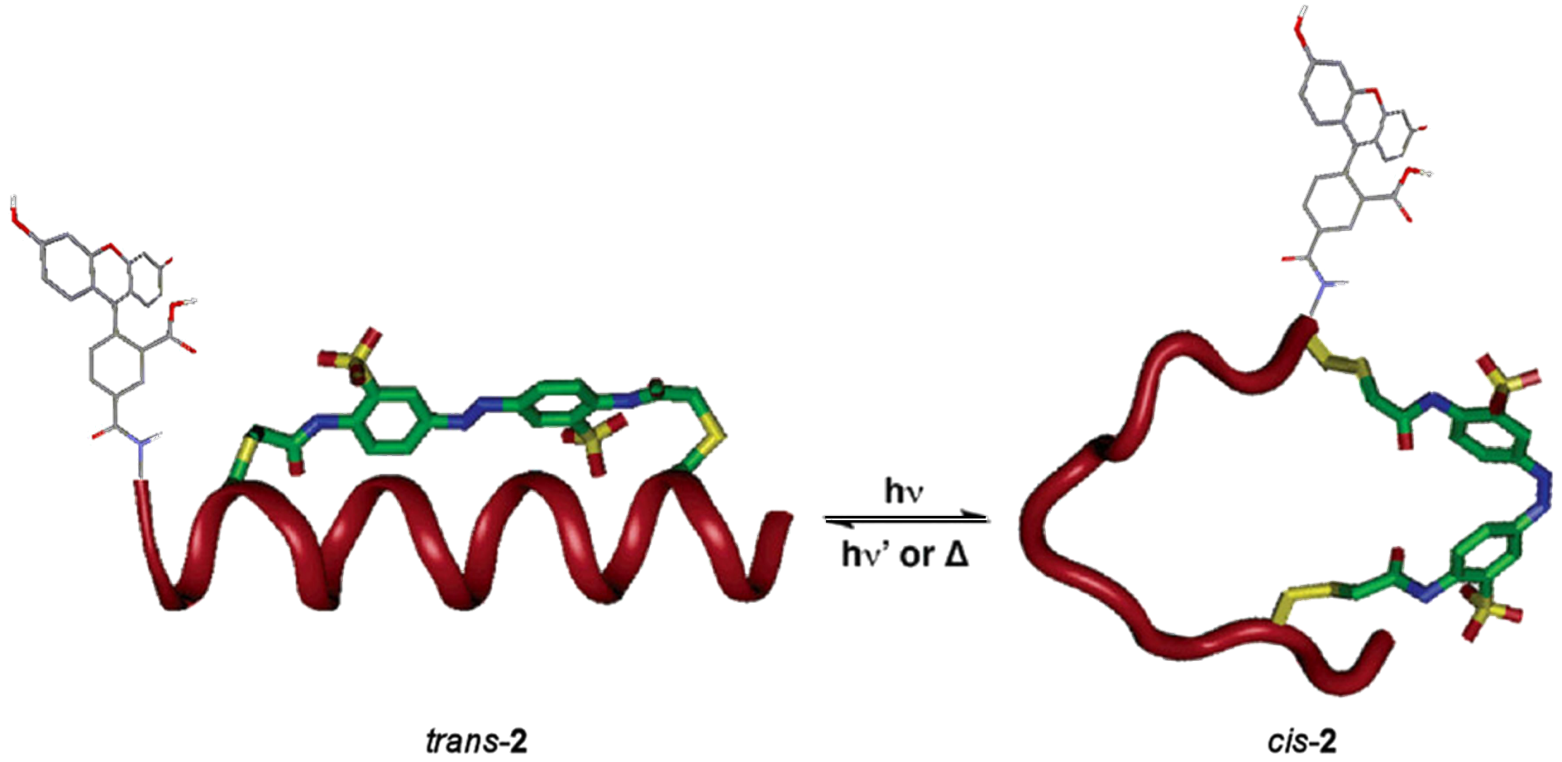
Photocontrol of the structure of the α-helix in the polypeptide azoderivative **2**. Reprinted (adapted) with permission from *J. Am. Chem. Soc.* **2005**, *127*, 15624–15625. Copyright (2005) American Chemical Society.

Another recent example was described by Woolley et al. [92–93]. They introduced an azobenzene moiety in a polypeptide to control the α-helical conformation and to have a synthetic tool that allows photomodulation of the very important conformation–interaction relationship in biological recognition. Peptides with pairs of cysteine residues were intramolecularly cross-linked with thiol reactive azobenzene-based photoswitches. Photoisomerization of the azobenzene changes the conformation of the peptide depending on the location of the cysteine. When the azo group of polypeptide **2** is in its *trans* form, it retains its affinity for DNA and its α-helical conformation. The photoisomerization leads to the *cis* isomer, which disrupts this helicity inhibiting the association with DNA. The photoreversion to the *trans* isomer recovers again the final conformation of the α-helix of DNA. In 2011, the same group carried out the attachment of a fluorescent dye close to the photoswitch giving rise to a fluorescence change upon isomerization. The introduction of azobenzene-modified biomolecules in zebrafish proved that the photochemistry of azobenzenes was similar in vivo and in vitro, and that appropriate azobenzenes could be stable in vivo for days (Figure 6) [94].

**Figure 7:**
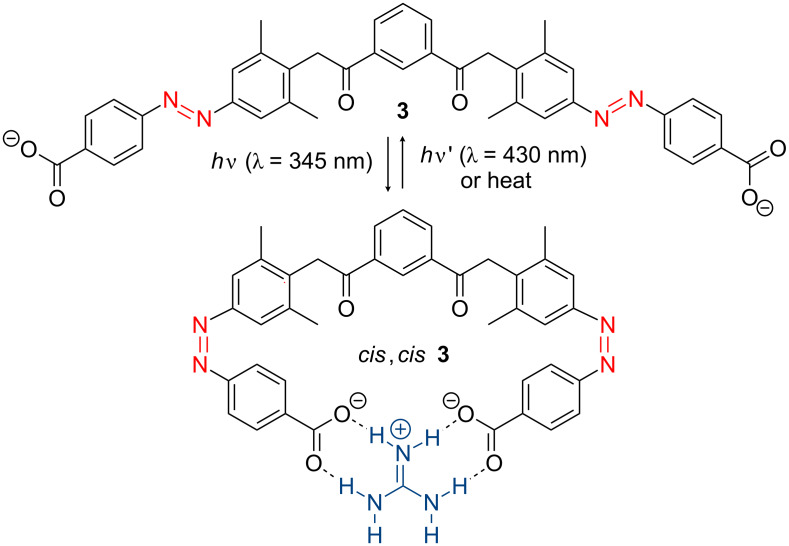
Recognition of a guanidinium ion by a *cis,cis*-bis-azo derivative **3**.

The photochromic properties of azobenzenes also find applications in “host–guest” recognition [95–96]. For example, the bis-azo compound **3** behaves as an excellent receptor of guanidinium ions by hydrogen-bonding interactions. The recognition is very effective when the azobenzene adopts the *cis* configuration (Figure 7) [95].

### Azobenzene-based molecular devices

**Figure 8:**
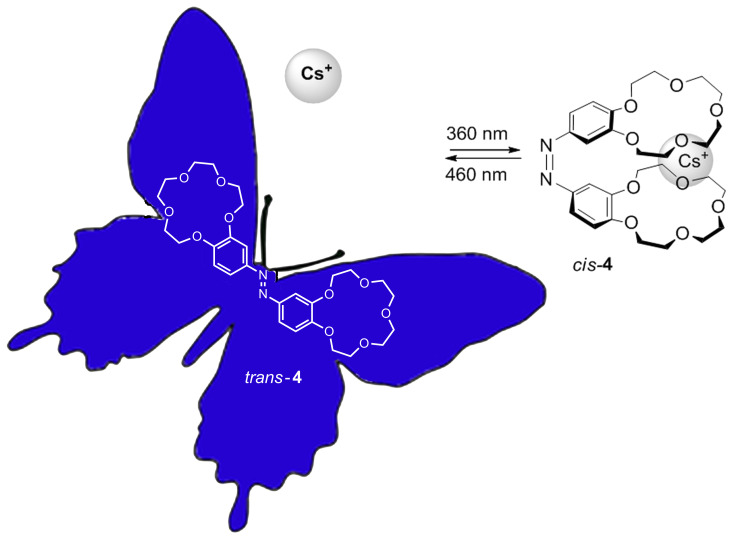
Recognition of cesium ions by *cis*-azo derivative **4**.

Among the systems with the inclusion and complexation properties of ions [97–100], several classes of compounds called azophanes, azocrowns [101–104], azocryptands, azocyclodextrins and azocalixarenes [105–107] have been described. The introduction of an azobenzene in these systems enables the photocontrol of the bonding properties of these molecules. The inclusion and complexation properties of some ions are more selective in one isomer that in the other. For example, the system of azocrown **4** shows a high selectivity for Rb^+^ and Cs^+^ ions [108]. The photoisomerization yields a similar motion to that of a butterfly, and only in the case of the *cis* isomer are the cations located between the two rings, yielding a “sandwich” structure (Figure 8). The ability of the azo compound **4** to remove cations from an aqueous solution increases in the order Na^+^< K^+^< Rb^+^< Cs^+^. According to these properties, compound **4** could be used as a selective transport system controlled by light.

**Figure 9:**
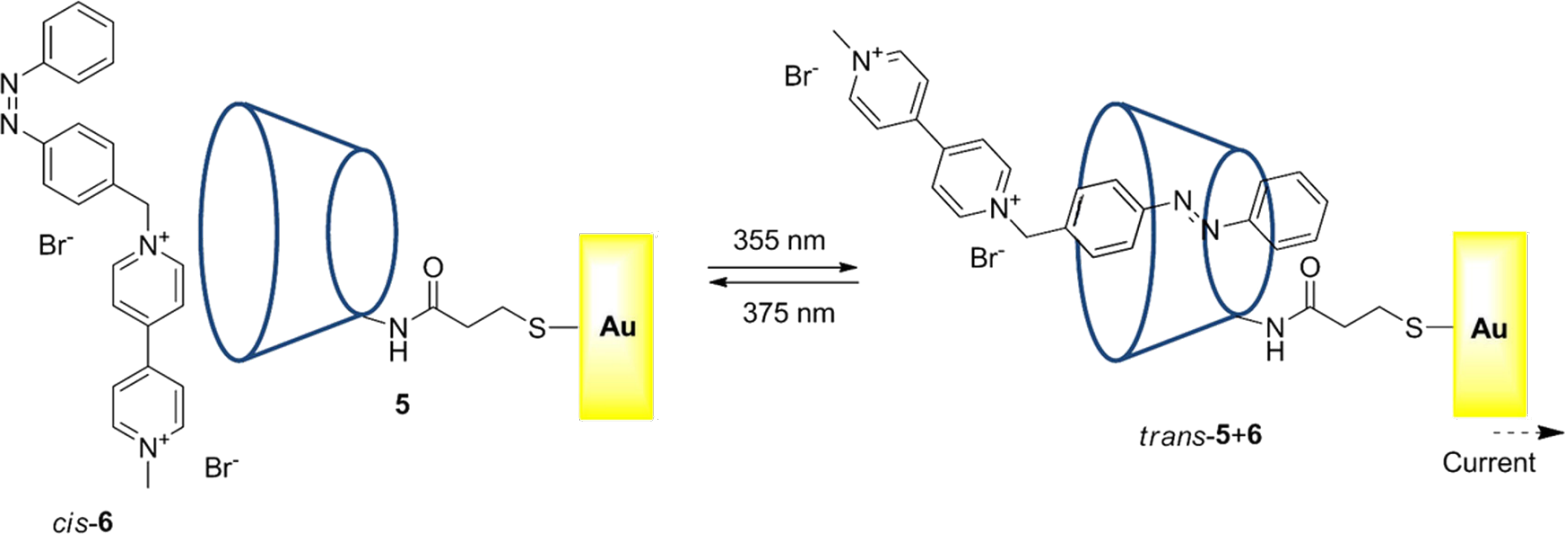
Photocontrolled formation of an inclusion complex of cyclodextrin *trans*-azo **5**+**6**.

The β-cyclodextrin-type structure, schematically represented in Figure 9 as **5**, acknowledges the bipyridinium fragment of diarylazobenzene **6** by formation of an inclusion complex of *trans*-**5**+**6**. This inclusion complex evolves in a reversible way when it is irradiated. The process is especially interesting for the translation of the optical signals recorded by the bipyridinium azobenzene **6** via a β-cyclodextrin single phase prepared on a gold electrode [109].

**Figure 10:**
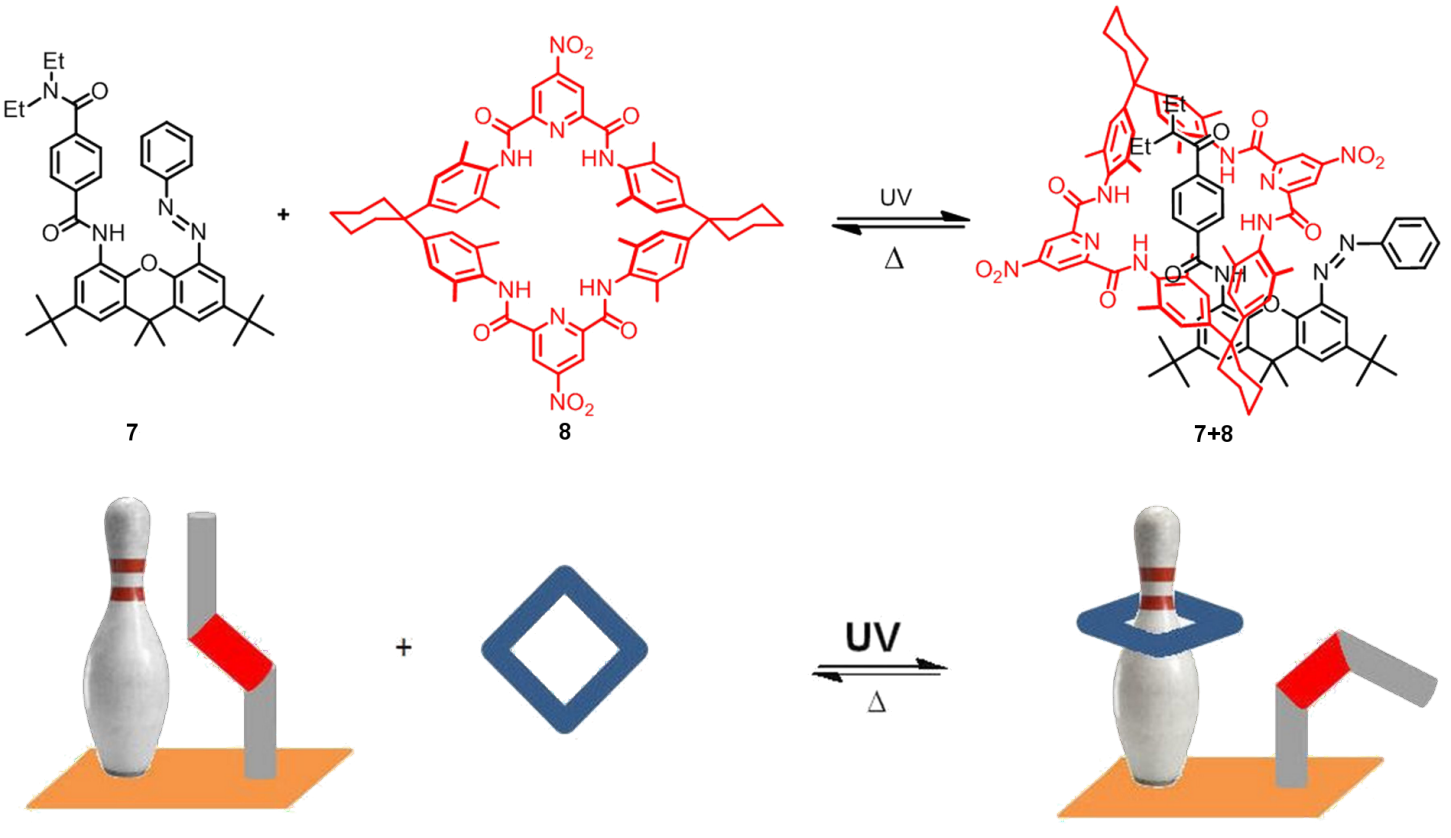
Pseudorotaxane-based molecular machine.

In 2003, a molecular machine based on a pseudorotaxane was described [110]. Assembly between **7** and **8** occurs only when the azobenzene **7** adopts the *cis* configuration. The pseudorotaxane **7**+**8** is disassembled into its two components when the isomerization to *trans*-azobenzene occurs by an external stimulus (Figure 10).

### Hinge molecular

**Figure 11:**
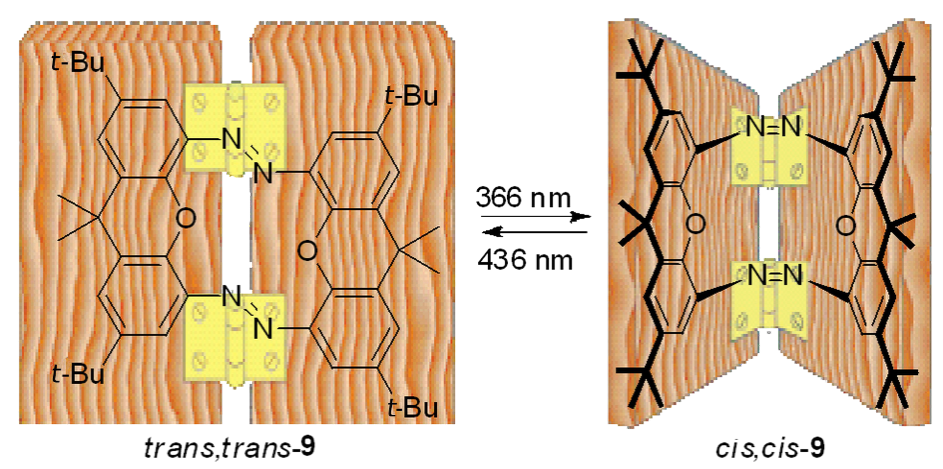
Molecular hinge. Reprinted (adapted) with permission from *Org. Lett.* **2004**, *6*, 2595–2598. Copyright (2004) American Chemical Society.

Tamaoki et al. [111] designed a molecular device capable of photo-emulating a hinge motion. This switch consists in two azobenzene units that share a fragment with two coplanar xanthenes (Figure 11). The photoisomerization of the system forces a molecular motion similar to a hinge, in which the two aromatic rings are arranged at an angle of 90°. The photoisomerization process involves three isomeric forms: (*trans*,*trans*), (*trans*,*cis*) and (*cis*,*cis*). The heats of formation of the three isomers were determined by ab initio quantum chemical calculations. The isomers (*trans*,*trans*) and (*cis*,*cis*) are 28 and 2.6 kcal∙mol^−1^ more stable than the intermediate isomer, respectively. The large energy difference between (*trans*,*trans*) and (*trans*,*cis*) isomers indicates the ring strain that exists in the (*trans*,*cis*) isomer, and the thermal isomerization from (*cis*,*cis*) to (*trans*,*cis*) is forbidden. The half-life of (*trans*,*cis*)-isomer is only 28 s at 23 °C. In these systems, in which the photochemical reaction intermediate has a short half-life and the final (*cis*,*cis*)-product is more stable than the intermediate, the photochemical yield is highly dependent on the used light intensity [112–114].

### Molecular threader

**Figure 12:**
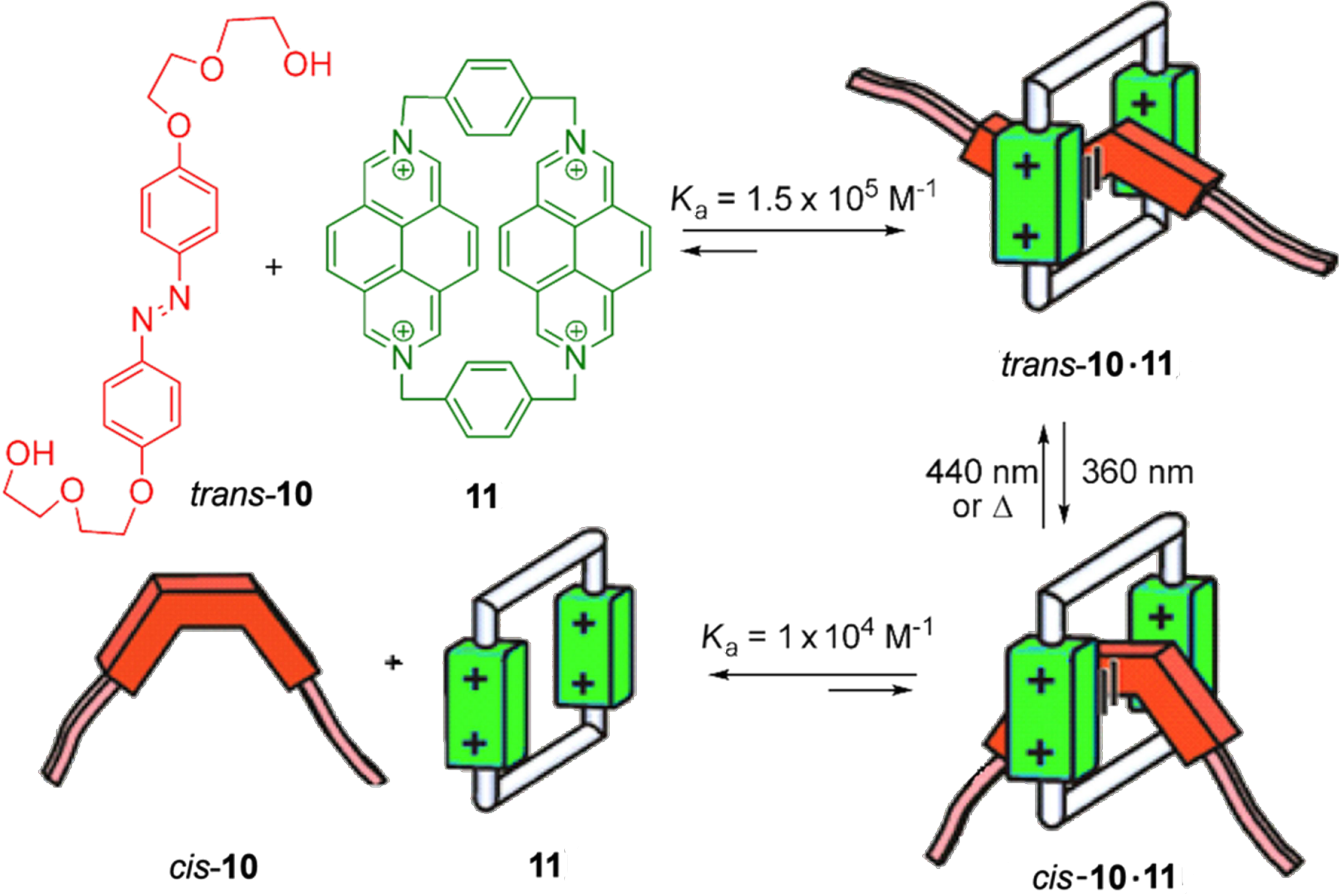
Molecular threader. Reprinted (adapted) with permission from *Acc. Chem. Res. ***2001**, *34*, 445–455. Copyright (2001) American Chemical Society.

Stoddart, Balzani et al. [115–116] created an intelligent molecular device (*trans*-**10**∙**11**) capable of moving within a cyclophane **11**, as a needle through a buttonhole (Figure 12). The interaction between the two systems is measured on the fluorescence emitted by the pyridinium salt free cyclophane. The azobenzene *trans*-**10** is conveniently replaced with electron donor units, so that when it is associated, as azo-**10**∙**11**, the fluorescence is completely inhibited by charge-transfer interactions. The photoexcitation carried out by irradiation with light of λ = 360 nm of a solution of *trans*-**10** and **11** causes a process of “unthreading”. The *cis* isomer **10** has a much weaker interaction with cyclophane **11**, and this fact is reflected in the large increase in fluorescence intensity of **11**. The *trans*-isomer **10** is regenerated when the mixture is left in the dark or irradiated with light of λ = 440 nm, and as a result becomes a “thread” in the cyclophane. In this way, the isomerization of the N=N double-bond type triggers a movement of threading/unthreading exclusively governed by light.

### Molecular scissors

**Figure 13:**
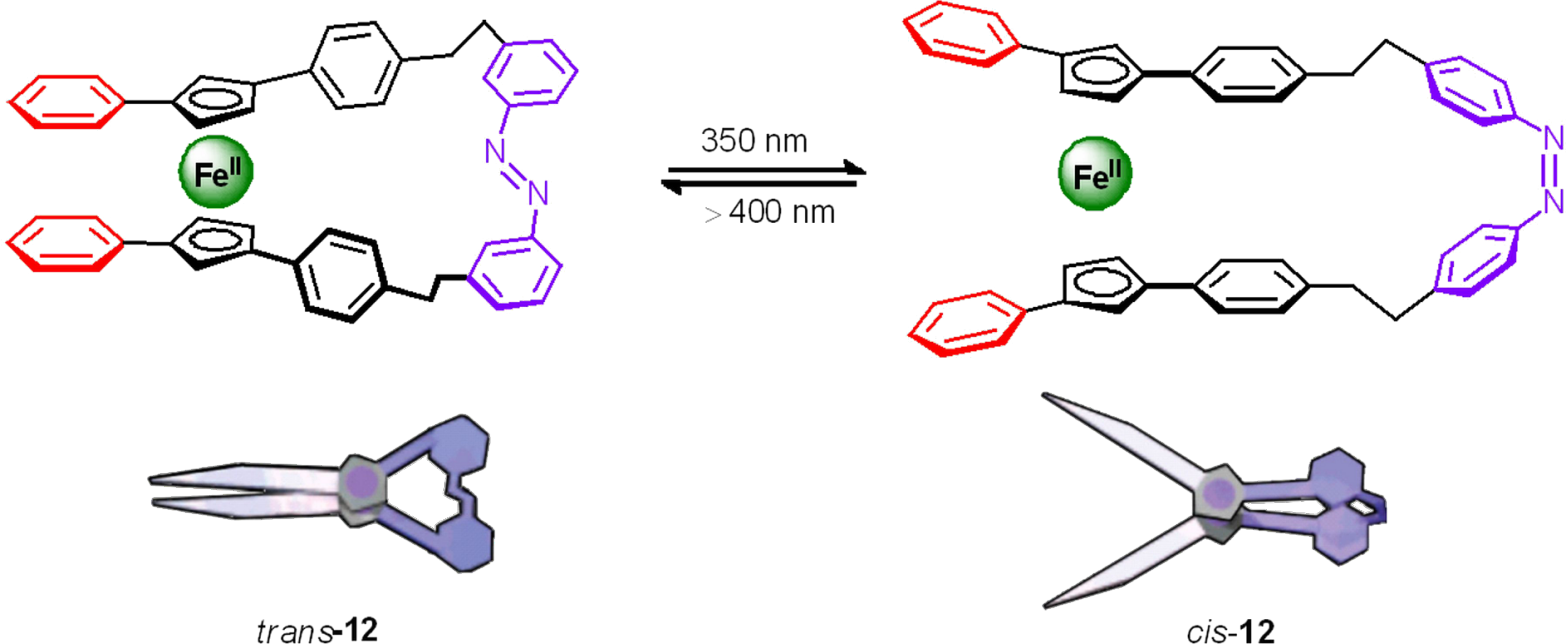
Molecular scissors based on azobenzene **12**. Reprinted (adapted) with permission from *J. Am. Chem. Soc.* **2003**, *125*, 5612–5613. Copyright (2003) American Chemical Society.

In 2003, Aida's group described a new generation of optical molecular devices composed of different kinds of organic systems interconnected through an azobenzene unit as the epicentre of the motion. First, a molecular switch was synthesized capable of making a motion similar to the opening and closing of scissors. This switch consists of a central unit of 1,1',3,3'-tetrasubstituted ferrocene, two phenyl groups as scissor blades and two phenylethylene groups as handles linked through an azobenzene [117–118]. The light irradiation of λ = 350 nm (180 min) leads to a mixture of isomers *trans*/*cis* 11:89, while exposure to visible light (λ > 400 nm, 15 min) again enriches 46% of the *trans* isomer. The molecular motion was studied by circular dichroism (CD), ^1^H NMR and DFT calculations confirming that the change in the configuration of the N=N double bond modifies the initial position of the ferrocene resulting in an opening (*cis*) and closing (*trans*) of the “blades” of the phenyl group (Figure 13). The angle between the two phenyl groups is altered from about 9° upon closing of the “scissors” (*trans*-**12**) to more than 58° when it opens (*cis*-**12**).

### Molecular pedals

**Figure 14:**
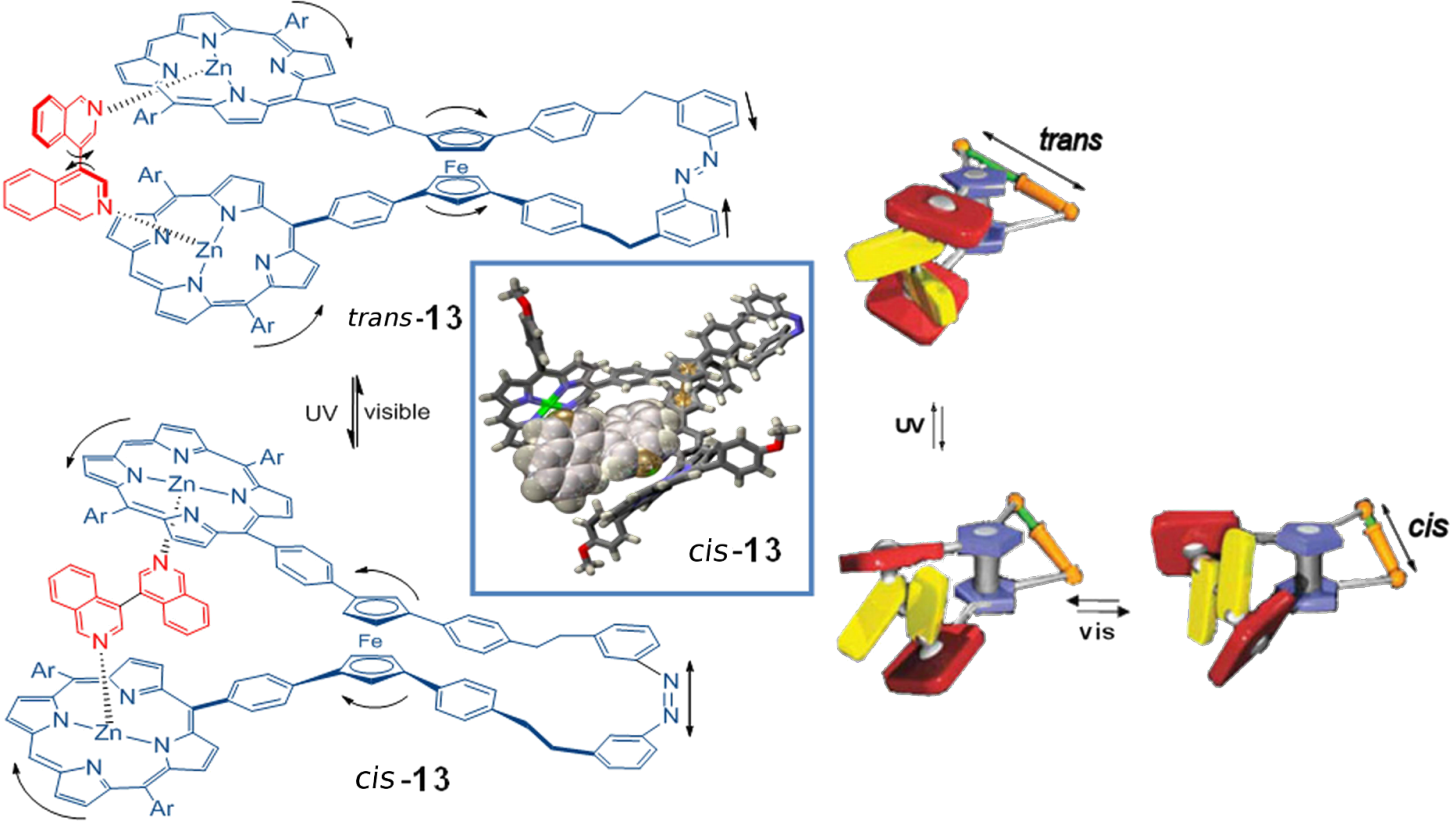
Molecular pedals. Reprinted by permission from Macmillan Publishers Ltd: *Nature*, **2006**, *440*, 512–515, copyright (2006).

In 2006 [119–120], the same authors described a more complex system that included two terminal units of porphyrin–Zn noncovalently associated to a host molecule of bis-isoquinoline **13** (Figure 14). The exposure of azo derivative **13** to light of λ = 350 ± 10 nm leads to a mixture of isomers *trans*/*cis* 22:78. Irradiation of this mixture of isomers at λ > 420 nm returns the system to an enrichment of the *trans* isomer (63%).

The study of the photoisomerization process of **13** revealed that the configurational change of the azobenzene unit causes a sequence of molecular motions of the units connected to it. The ferrocene unit rapidly responds by turning, which in turn induces an opening motion that distances the porphyrin units, causing a mechanical spin rotation in the bis-isoquinoline molecule similar to a pedal. This device is effective if the porphyrin–Zn and bis-isoquinoline units remain associated during the photoisomerization, i.e., the dynamics of dissociation between these units is slower than the photoinduced movement itself. In this case, the dissociation constant is six orders of magnitude slower than the *trans*–*cis* photoisomerization, which ensures that the bis-isoquinoline unit is coordinated to azocompound **13** during the isomerization process of the azobenzene unit.

### Nanovehicle

**Figure 15:**
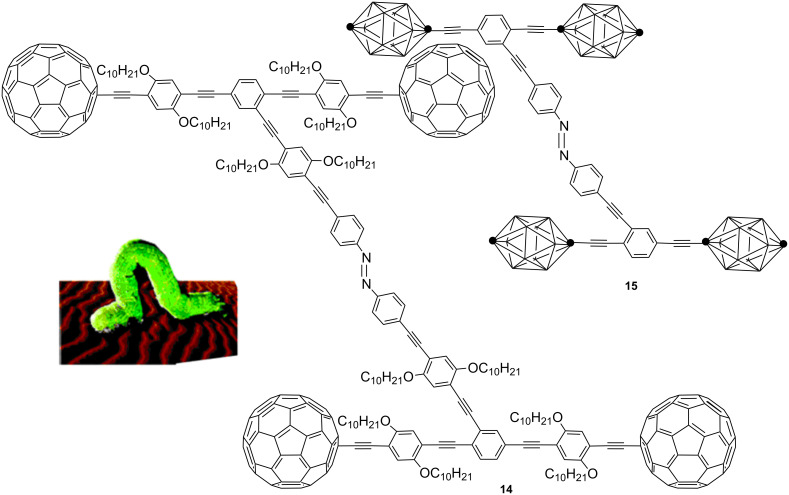
Design of nanovehicles based on azo structures. Reprinted (adapted) with permission from *Org. Lett.* **2008**, *10*, 897–900. Copyright (2008) American Chemical Society.

One proposed mechanism for the isomerization of azobenzene suggests that photoinduced *trans*–*cis* isomerization passes through a rotating mechanism, while the thermal *cis*–*trans* reisomerization follows an inversion mechanism [121]. The combination of both processes (photochemical and thermal) could lead to opening (*trans*) and closing (*cis*) mechanical motion accompanied by a translational motion. Based on this mechanistic hypothesis, Tour et al. created a branched azobenzene structure to realize a nanovehicle able to move like a caterpillar (Figure 15) [122–123].

The system consists of three parts: A central azobenzene, a rigid frame composed of oligo(phenylacetylenes), which in turn are anchored to azobenzene through *para* positions, and wheels based on fullerene C_60_ (azo-**14**) or *p*-carboranes (azo-**15**). Photoisomerization studies suggest that only the azobenzene system with *p*-carborane wheels (azo-**15**) may be useful as a molecular switch, because the photoisomerization of azo-fullerene **14** leads to only 8% of the *cis*-isomer. Although, the quantum yield obtained for *cis*-**14** is not very high, this proportion is significant, given the speed with which energy transfer to the fullerene unit occurs [124]. In the case of azo-*p*-carborane **15**, irradiation at λ = 365 nm for 10 min leads to 24% of the *cis* isomer. The photochemical (λ > 495 nm, 5 min) or thermal (heating to 40 °C, 15 min) reisomerization recovers the initial state. After these preliminary results it remains to be demonstrated whether the molecular motion of the device **15** achieves the expectations of the authors, to prove the usefulness of the device.

### Molecular driving force

**Figure 16:**
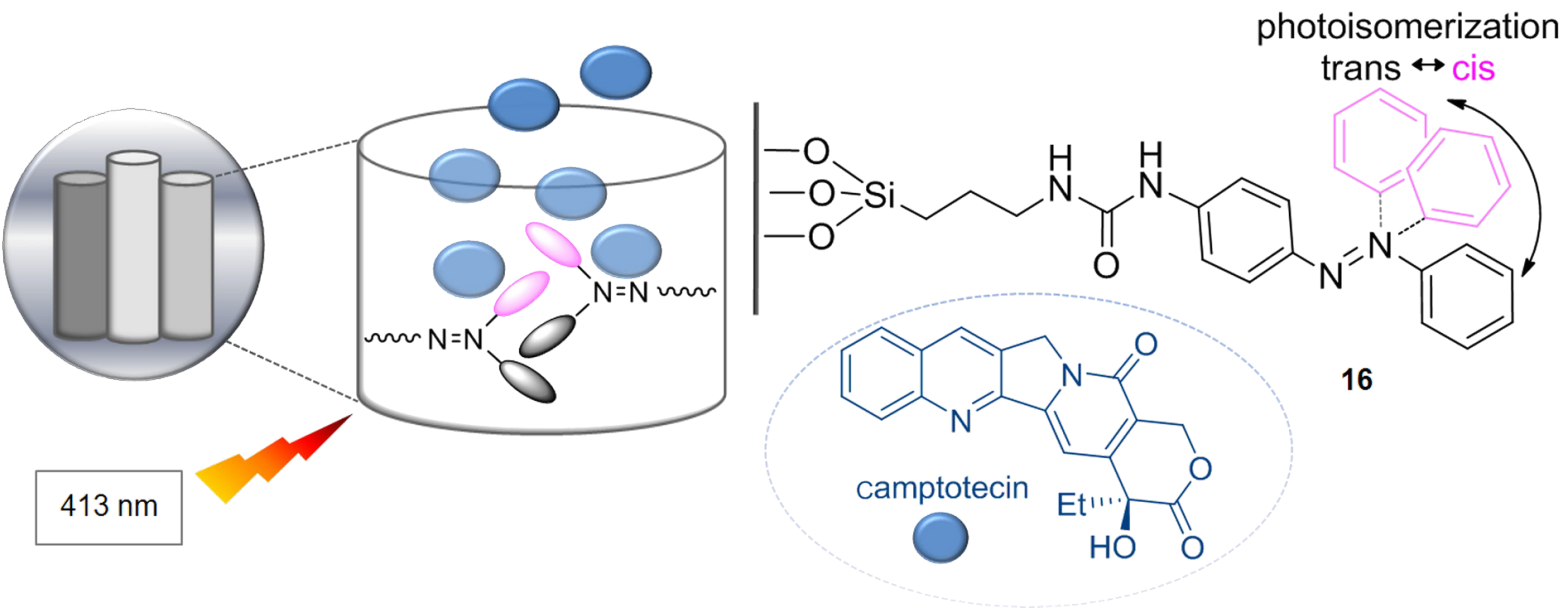
Light-activated mesostructured silica nanoparticles (LAMs).

One of the more attractive and interesting applications of the isomerization processes of azobenzenes is their use as nanoimpeller-controlled drug release devices. The idea is to anchor a functionalized azobenzene inside the silica nanoparticle, thereby forming light-activated mesostructured silica nanoparticles. The azobenzene **16** is anchored to the particle wall while the other extreme is free (Figure 16) [125–127].

These nanoparticles have pores capable of holding other molecules. The isomerization process of azobenzene generates a molecular flapping and the molecules can be expelled outside in a photoinduced way. The morphology of the light-activated mesostructured silica nanoparticles (LAMs) is evaluated by scanning electron microscopy (SEM), electronic transmission images (TEM), UV–vis and X-ray analysis, setting a pore diameter of 1.9 ± 0.1 nm, a volume of 0.248 cm^−3^∙g^−1^ and a surface area of 621.19 m^2^∙g^−1^. The nanoparticles, which contain 2.4% by weight of azobenzene, are treated with camptothecin (CPT), a drug used in the treatment of cancer, which is housed inside. The LAMs (CPT) were incubated for 3 h with cancer cells in darkness. These cells are irradiated for 5 min at 0.1 W∙cm^−2^ at λ = 413 nm, where both isomers have the same extinction coefficient promoting a continuous exchange between both *trans*–*cis* isomers, and then they were incubated again in the dark for 48 hours. This experiment shows that the azobenzene units located within the LAMs (CPT) act as promoters (Figure 16), releasing the drug (CPT) only after irradiation of the nanoparticle with a light at a certain wavelength, hence resulting in cell death. The number of released molecules can be controlled depending of the light intensity and irradiation time. On the other hand, the camptothecin, in the absence of light, stays inside the nanoparticles and the cells remain intact. Control experiments with cells lacking the nanoparticles revealed that irradiation at 413 nm does not affect cell survival, and similarly irradiation of incubated cells with LAMs not containing CPT, did not lead to cell death, thus confirming the biocompatibility of the LAMs with the cells.

### Molecular lift

**Figure 17:**
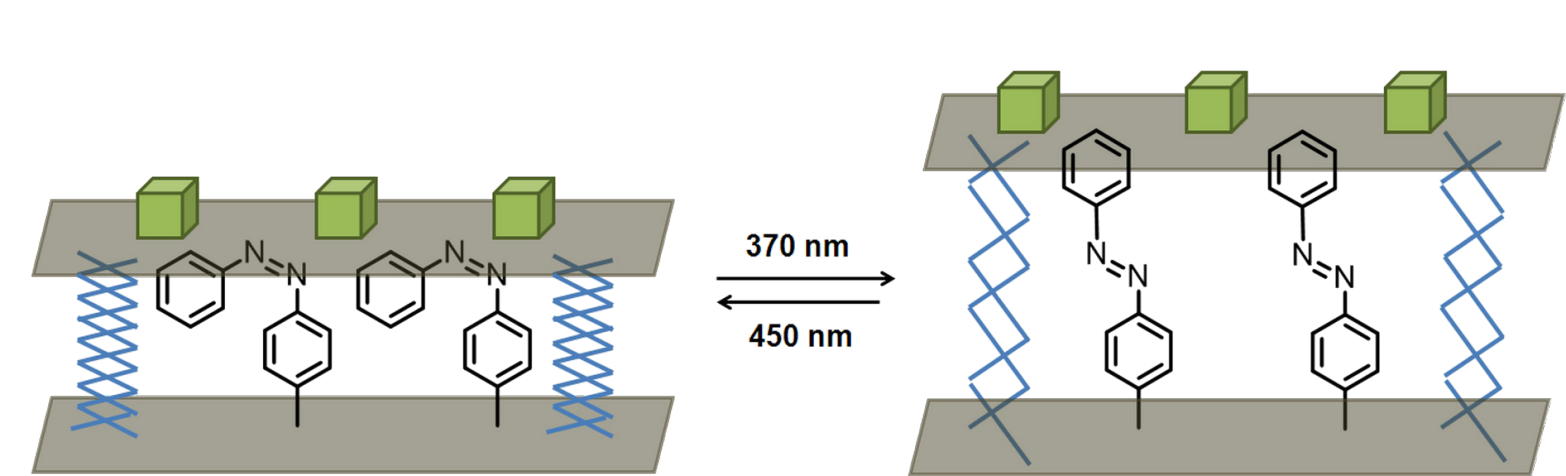
Molecular lift.

The individual molecular motion of azobenzene in the *cis*–*trans* isomerization process can be amplified when the azobenzene is anchored to a more complex system [128]. The cooperative combination of each individual photoisomerization can increase the dynamic response if the azobenzenes are self-assembling, generating an uniform motion [129]. An illustrative example is given in Figure 17, in this case one of the azobenzene rings has a *p*-mercaptophenyl group through which it is associated with an Au(111) layer [130]. All the azobenzenes are oriented and form self-assembled monolayers (SAMs). The *trans*–*cis* isomerization process of the azobenzene unit placed in the metal layer takes place with excellent yield (88–98% *cis* isomer). This is particularly relevant for future applications in the design of devices for information-storage-based photochromic systems [131]. The photo-reversal of SAMs also proceeds with excellent yield (94–100%). The structural difference (*d*_trans_–*d*_cis_) between both isomers is approximately 7 Å.

By scanning tunnelling microscopy (STM), the authors determined the surface density of SAM_azo_, and the force exerted in the photoisomerization molecular motion of all the azobenzenes in the SAM_azo_ was calculated. The individual photomovement of each azobenzene results in a collective structural change in a certain direction. This cooperative molecular motion of SAM_azo_ acts as a molecular lift capable of lifting one Hg drop deposited on the monolayer of azobenzenes. Furthermore, this device acts as a photoswitch of the current between the Au(111) layer and the Hg drop. A significant increase of the current density of about one order of magnitude occurs under irradiation at λ = 370 nm, and the corresponding decrease is produced in a reversible way when the azobenzene is irradiated at λ = 450 nm.

### Molecular sunflower

**Figure 18:**
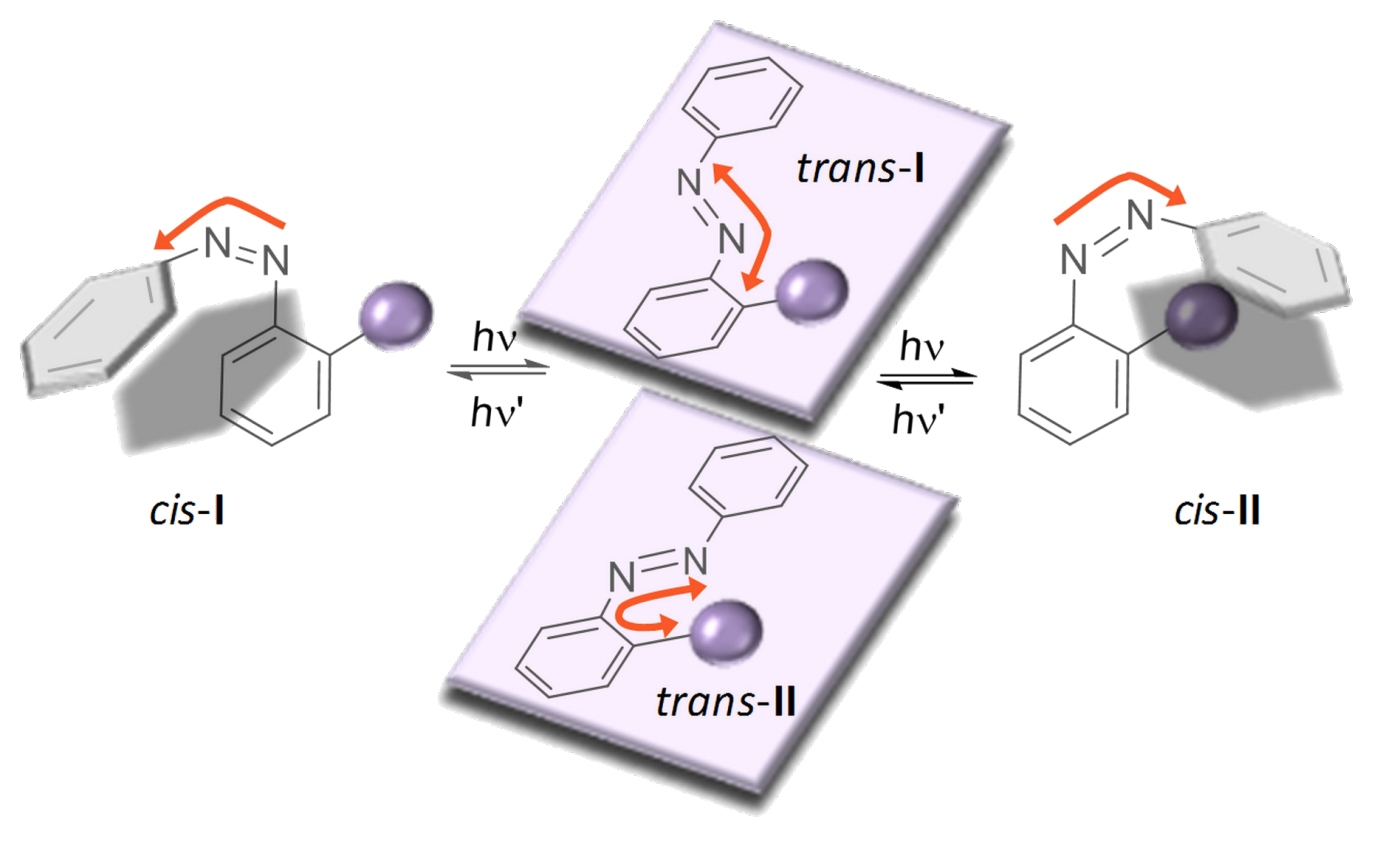
Conformational considerations in mono-*ortho*-substituted azobenzenes.

When an azobenzene is differently substituted in the *ortho* or *meta* positions, the corresponding *trans* and *cis* isomers can adopt different conformations. A simple example is illustrated in Figure 18 for mono*-ortho*-substituted azobenzene. In the *trans* isomer, the azo group is oriented as far as possible away from the substituent in the *ortho* position (*trans*-**I** form), or placed next to it (*trans*-**II**). Similarly, motion of the aromatic rings accompanying the photoisomerization process can also lead to several *cis* conformers (*cis*-**I** and *cis*-**II**). The electronic nature and steric bulk of the substituents of the aromatic rings can be a key factor in favouring one type of conformation in the azocompounds. Considering the relationship between the molecular conformation and biological recognition, it is particularly interesting to design azo devices that allow control, by an external stimulus, of the configuration of the N=N double bond, and also to define a specific orientation or conformation of each stereoisomer (*trans* or *cis*).

**Scheme 1:**
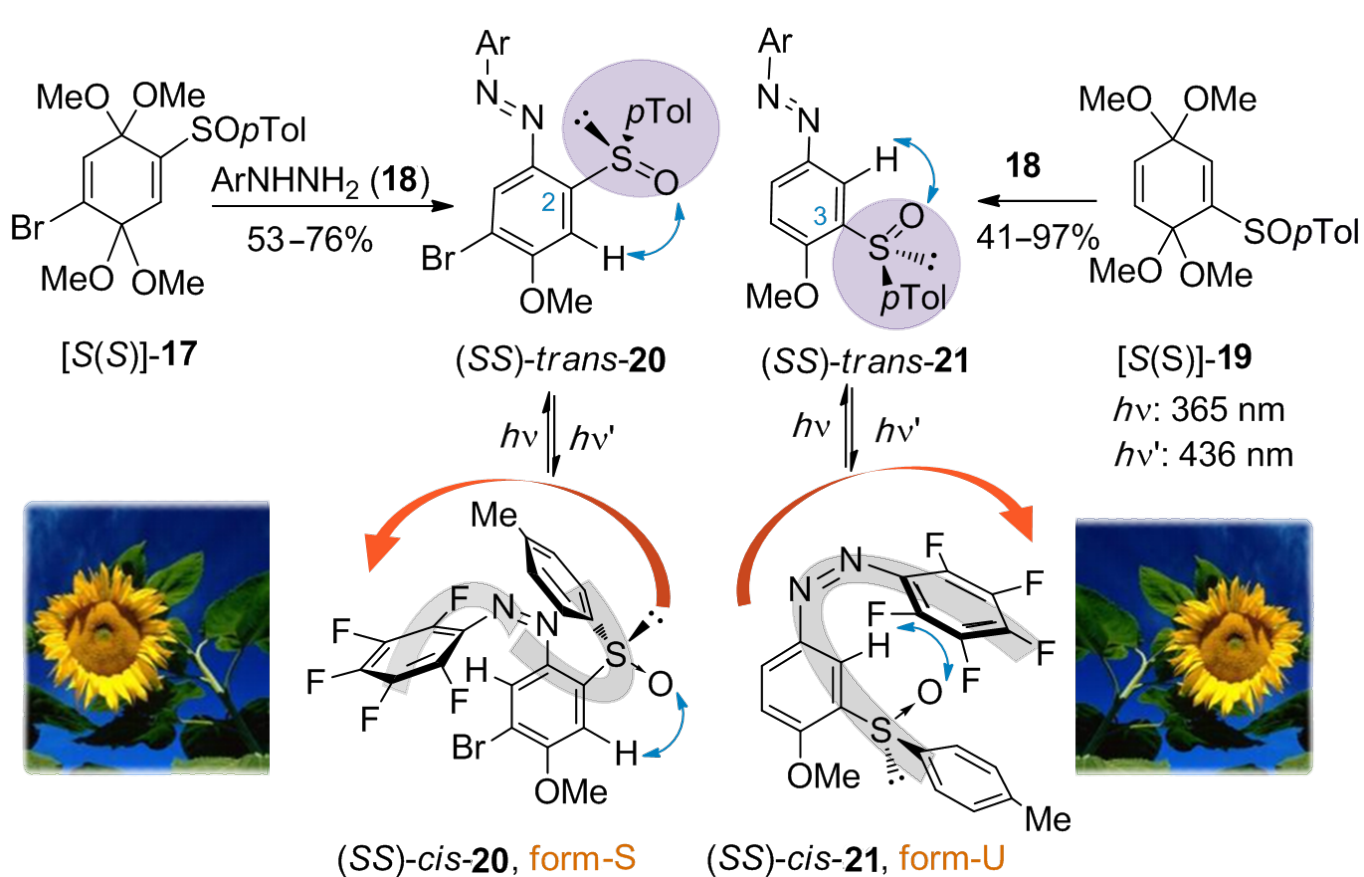
Synthesis and photoisomerization of sulfinyl azobenzenes. Reprinted (adapted) with permission from *J. Am. Chem. Soc.* **2007**, *129*, 7089–7100. Copyright (2007) American Chemical Society.

Recently, Carreño et al. [132–133] synthesized different enantiomerically pure sulfinyl azobenzenes. The sulfinyl group is a key component in the design of a molecular sunflower, a device that by means of light can undergo phototropism with a given direction. The enantiomerically pure 2- and 3-sulfinyl azo compounds are obtained with excellent regioselectivities by using a new and simple method for the synthesis of aromatic azobenzenes based on the treatment of quinone bisacetals **17** and **19** with different arylhydrazines **18** [134]. In both cases, the sulfoxide group preferentially adopts a rigid S-*cis* conformation [135], situating the sulfinylic oxygen in 1,3-parallel arrangement with the neighbouring hydrogen (blue arrow, Scheme 1). This arrangement is essential to force a specific final conformation of the azocompound.

Irradiation with light of λ = 365 nm results in 33–75% of *cis*-isomer **20** and 84–99% of *cis*-**21**. The photochemical reisomerization (λ = 436 nm) recovers the initial state in both sets of sulfinyl derivatives. The study of the photochromic properties of enantiopure azocompounds **20** and **21** by using standard techniques (UV–vis, circular dichroism, chiral HPLC and NMR) has established that the chiral optical response differs greatly depending on the position of the sulfoxide group (C-2 or C-3). *Cis* isomers in both *p*-tolylsulfinyl azocompounds show an opposite arrangement of substituents around the N=N group, with an S-shaped structure for *cis*-**20** or a U-shaped structure for *cis*-**21**. The conformational rigidity of the chiral sulfinyl group is the key to controlling the directionality of the molecular motion of photoisomerization. Thus, choosing the position of the sulfoxide group in the azobenzene (*ortho* or *meta* to N=N) by irradiation with light causes a determined geometric, conformational and rigid change, inducing a specific phototropism as in the stem of a sunflower.

### Photoactive Brønsted base

**Figure 19:**
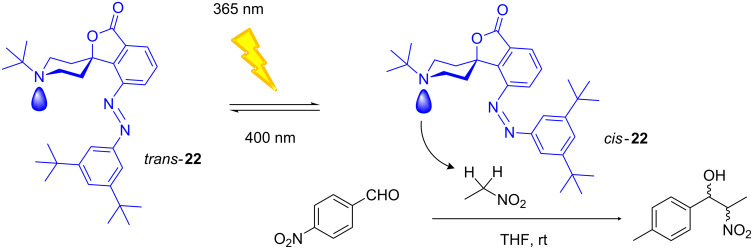
Photoisomerization of azocompound **22** and its application as a photobase catalyst.

The conformation of a molecule can have direct implications for its reactivity. Thus, the control of the conformation is the key to controlling its reactivity. The union of this concept with molecular switches has opened the door to the development of new photoreactive compounds in which the reactivity can be controlled by an external stimulus as a switch (on/off). Recently, Hecht et al. [136–137] designed a Brønsted base whose p*K*_a_ changes with light. The study focuses on the azobenzene **22**, which possesses, in an aromatic ring, a spirocyclic lactone fused to a conformationally restricted piperidine (Figure 19).

In the structure of the *trans* isomer **22**, the pair of unshared electrons of nitrogen is inaccessible. The *trans*–*cis* photoisomerization process changes the disposition of the aromatic rings, unlocking access to the basic center of the piperidine. This switch, of Brønsted base type, has been tested in the Henry reaction between *p*-nitrobenzaldehyde and nitroethane ensuring that only the *cis* isomer is able to catalyze the reaction.

### Photodirected azo polymers

**Figure 20:**
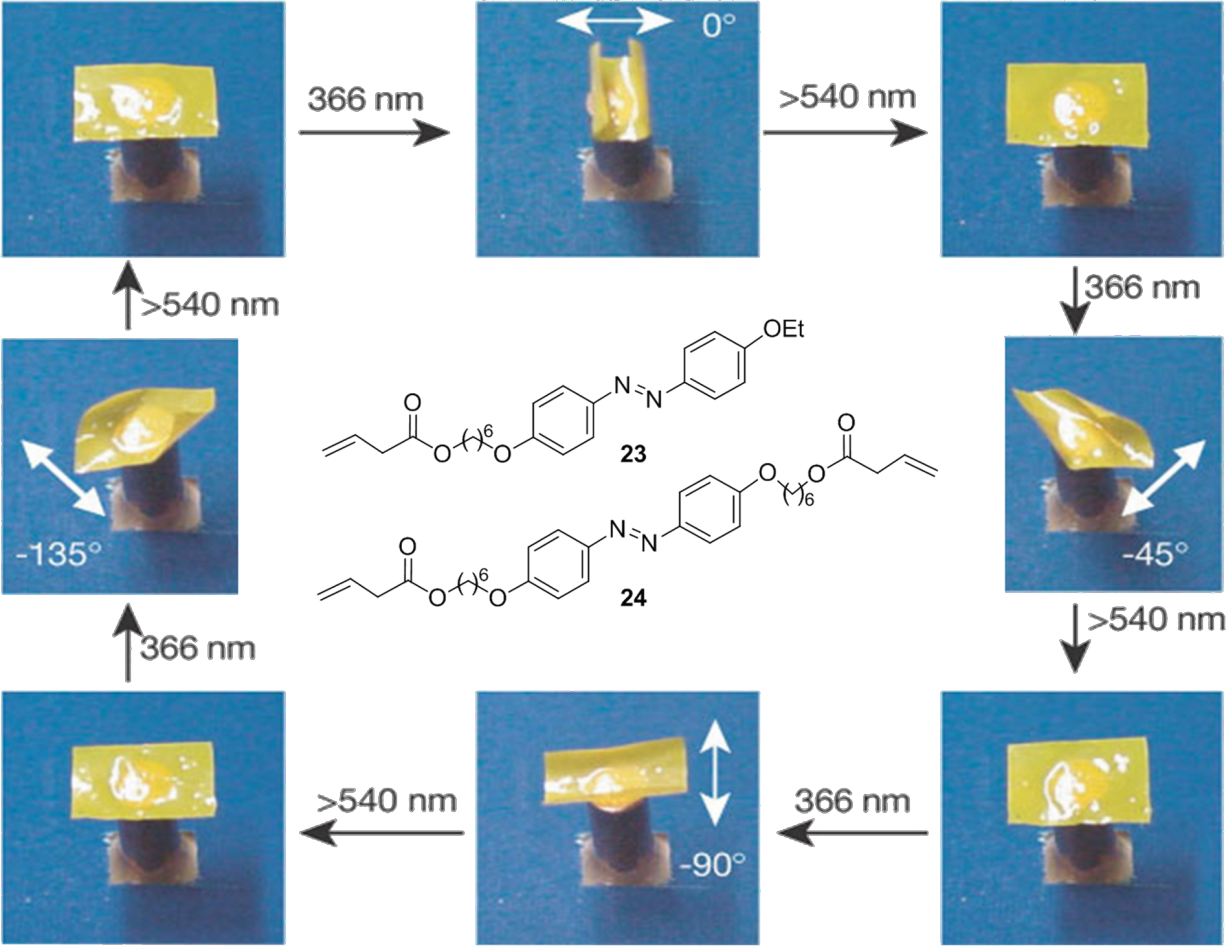
Effect of irradiation with linearly polarized light on azo-LCEs. Reprinted by permission from Macmillan Publishers Ltd: *Nature*, **2003**, *425*, 145–146, copyright (2003).

A spectacular example of the molecular motion of photoisomerization is shown in Figure 20. The irradiation of polymers containing light-sensitive molecules such as azobenzenes can lead to a photocontraction of the polymer, converting luminescent energy to mechanical energy. Ikeda et al. [37] demonstrated how the irradiation of a liquid-crystal elastomer (LCE) of azobenzenes with linearly polarized light is able to collapse and expand the LCE films (yellow sheet in Figure 20) in a certain direction.

The film is obtained by thermal polymerization of the azocompound **23** (monomer), by using the diacrylate derivative **24** as a crosslinking agent. The azo-LCEs comprise a polydomain with the characteristics of a liquid crystal, formed by many microsized domains of azobenzene aligned in the same direction. Although macroscopically the direction of alignment is random, under irradiation with linearly polarized light the selective absorption of the light by the azobenzene causes a collective management of all microdomains, such that the orientation of the fold is governed by the direction of the linearly polarized light source (white arrow in Figure 20). The consecutive irradiation with λ = 366 nm at 0°, −45°, −90° or −135° following by irradiation at λ = 540 nm produces the contraction and expansion of the film in the clockwise direction. Recently, this liquid-crystal elastomer was used in developing the first plastic photomechanical motor capable of converting light into mechanical energy without any battery or power source [47].

### Bistable memory device

**Figure 21:**
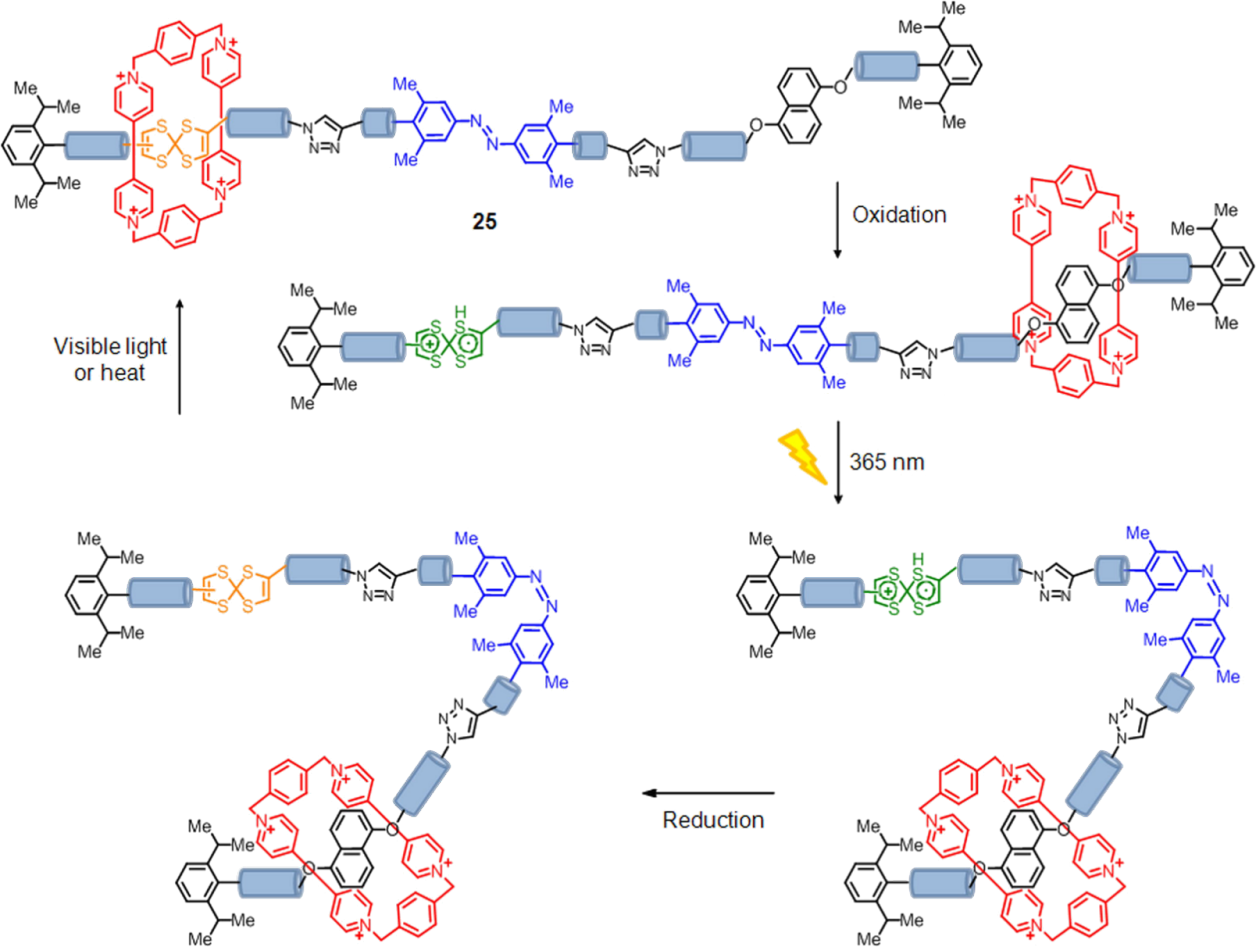
Chemically and photochemically triggered memory switching cycle of the [2]rotaxane **25**.

Recently, Stoddart and Venturi's group described a molecular switch using a [2]rotaxane **25**, which undergoes mechanical movements triggered by redox processes and can be switched between two thermodynamically stable conformations [138]. The energy barriers between these conformations can be controlled kinetically by photochemical modulation. The ring component of the [2]rotaxane is cyclobis(paraquat-*p*-phenylene) and the dumbbell is comprised of a tetrathiafulvalene unit and a 1,5-dioxynaphthalene as π-electron-donating recognition sites, and a photoactive unit of 3,5,3',5'-tetramethylazobenzene, which by irradiation of light can be switched between its *cis* and *trans* conformations (Figure 21). Probably, this structure is a good candidate for use in the design of engineered test devices. Data can be written on the rotaxane when the units of tetrathiafulvalene are oxidized and then blocked in the *trans*–*cis* photoisomerization process, on the azobenzene fragment. After writing the information, the oxidized species can be reduced to the original form without loss of data. The data is stored until irradiation of the azobenzene fragment allows the reopening of the azobenzene gate.

### Unidirectional photoisomerization

**Figure 22:**
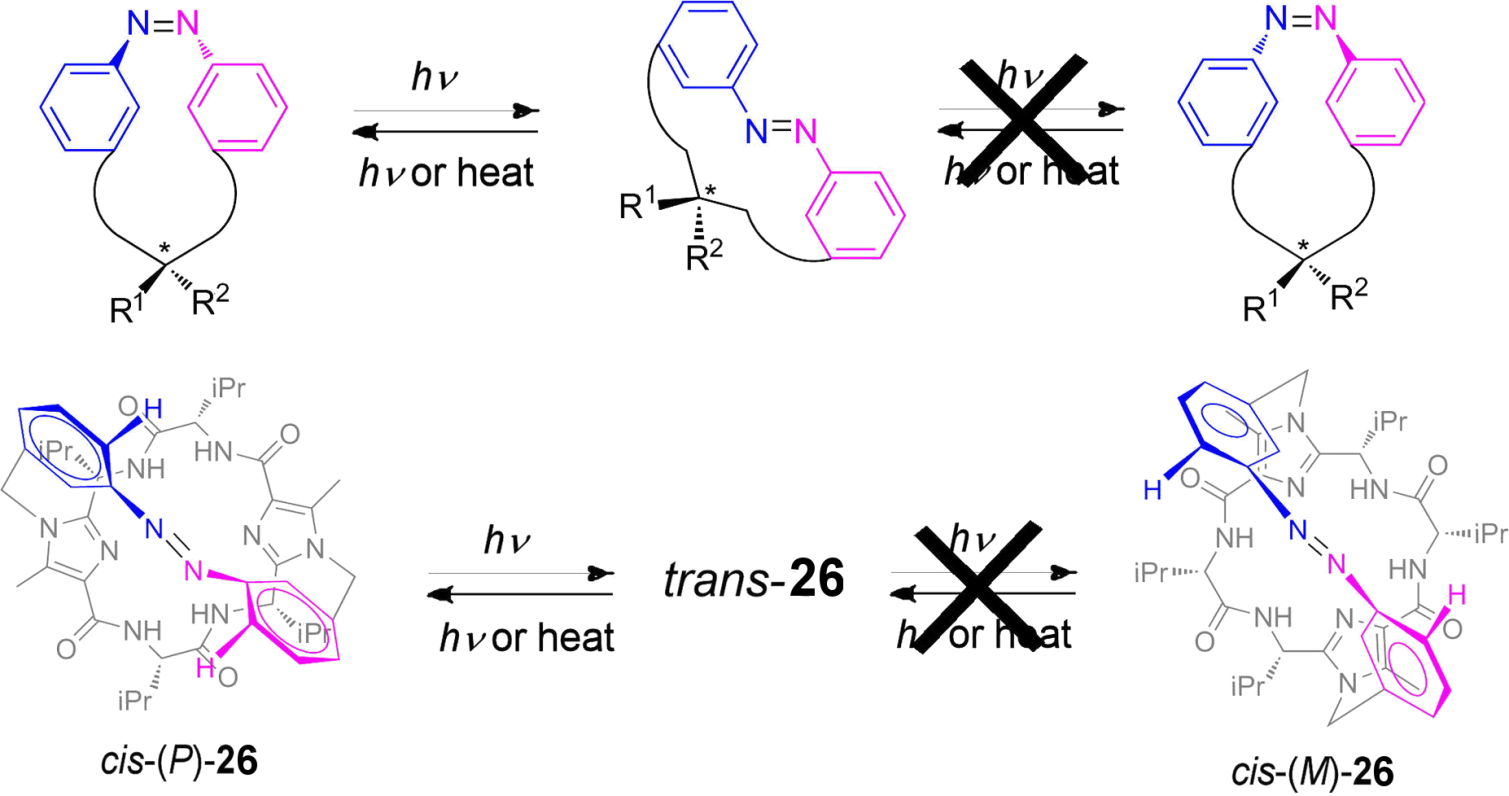
Unidirectional photoisomerization process of the azobenzene **26**.

The process, *trans* to *cis* isomerization, generates helicoidal chirality, such that the isomer can adopt a helicoidal geometry with *P* or *M* chirality. The configurational stability of *cis*-azobenzenes depends of the interconversion barrier between the *cis*-(*P*) and *cis*-(*M*) isomers. The size and the electronic nature of the substituents present in the systems are the factors that are more influential on the energetic barrier. Haberhauer et al. described the unidirectional photoisomerization process of azobenzene **26** [139]. The irradiation with light of the achiral *trans* isomer gives rise to the *cis* isomer with *P* helicity. A chiral clamp was synthesized, by anchoring a chiral cyclic imidazole peptide to both aromatic rings of azobenzene. The system is flexible enough to allow the isomerization between the *trans* and *cis* isomer but in turn destabilizes one of the helices of the *cis* isomer, and only one *cis* isomer (*P*) is present in solution (Figure 22).

## Conclusion

Azobenzene is one of the most used systems in the design of molecular photoswitches. The synthesis is very easy and their photochromic properties are very interesting. An external stimulus, normally light irradiation at a certain wavelength, causes a fluctuation between the *cis*–*trans* isomeric species. This isomerization is reversible, photochemically and thermally. The molecular motion that occurs in the isomerization process has facilitated the development of molecular devices. Today, researchers continue to develop synthetic supplements to improve the properties of azobenzenes towards the development of more efficient devices that control the isomerization and orientation of the azobenzene, shifting the photoisomerization process to other concepts of chemistry, from the development of photoreactive compounds or photomechanical materials and even bioincorporation of azobenzenes, to more complex systems that allow a greater understanding, such as the photocontrol of biological dynamic mechanisms.
